# Is there a potential link between keratoconus and autism spectrum disorders? A case report and literature review

**DOI:** 10.1097/MD.0000000000020247

**Published:** 2020-05-29

**Authors:** Svetlana Stanojlovic, Milica Pejovic Milovancevic, Branislav Stankovic

**Affiliations:** a Faculty of Medicine, University of Belgrade; b Clinic for Eye Diseases, Clinical Centre of Serbia; c Institute of Mental Health, Belgrade, Serbia.

**Keywords:** autism spectrum disorder, corneal cross-linking, eye rubbing, keratoconus, progression

## Abstract

**Rationale:**

Eye rubbing (ER) is a proven factor that can trigger the onset and progression of keratoconus (KC). Apart from allergy, ER is a repetitive motor stereotypy. Eye rubbing is frequently observed in children with autism spectrum disorders (ASDs) and in individuals who may be at risk for developing KC. We present a child with ASD who developed progressive KC following standard corneal cross-linking (CXL), most likely because of abnormal ER associated with allergy and repetitive behavior due to ASD symptoms.

**Patient concerns:**

A 14-year-old boy was referred to our clinic because of asymmetric visual acuity reduction.

**Diagnosis:**

The child was diagnosed as having keratoconus. He had a strong ER habit. The child had been previously diagnosed as having ASD.

**Interventions:**

Corneal cross-linking was performed in both the eyes. On account of keratoconus progression, most likely associated with persistent ER habit, he was retreated with CXL in the right eye. Behavioral modification intervention for ER habit reversal was also applied.

**Outcomes:**

Corneal cross-linking in combination with behavioral modification intervention for ER habit reversal prevented further KC progression.

**Lessons:**

Behavioral interventions are likely to provide positive results in an ER habit reversal in children with ASD. Keratoconus treatment with CXL combined with behavioral management for ER reversal seemed effective in halting keratoconus progression in a young patient with ASD.

## 1 Introduction

Autism spectrum disorders (ASDs) are a group of developmental disorders with increasing prevalence worldwide. A surveillance study identified 1 in 59 children as having ASD.^[1]^ Autism spectrum disorder is interestingly up to 4 times more often diagnosed in male individuals.^[1]^ The main clinical manifestations of ASD are persistent deficits in social communication and repetitive and/or restricted patterns of behaviors.^[2]^ For a clinician, the assessment and management of behavioral problems in people with ASD present a continuous challenge because of the condition per se and because of frequent comorbidities. Ophthalmologic disorders exist in 40% of children with ASD, and 29% of these children have significant refractive errors.^[3]^ Corneal astigmatism is interestingly significantly more prevalent among children with ASD than among the typically developed population group (46.2% and 25.6%, respectively).^[4]^

Keratoconus (KC) is characterized by decreased structural stability of corneal tissue that results in localized corneal thinning.^[5]^ The progression of KC induces irregular astigmatism and myopia; these disorders can cause significant visual impairment.^[5]^ Accurate recognition of early ectatic changes and KC progression has become more important with the development of corneal cross-linking (CXL).^[6,7]^ Photoxidative CXL between corneal collagen fibers is achieved using riboflavin (vitamin B_2_) as a photosensitizer and ultraviolet (UV)-A light.^[7]^ The treatment is aimed at increasing biomechanical stiffening of the cornea to halt or slow the progression of KC.

The Global Consensus on Keratoconus and Ectatic Diseases defines KC as multifactorial disease with genetic, environmental, and biomechanical components.^[6]^ Whether atopy per se or eye rubbing (ER) secondary to allergy has a casual link with KC remains unclear. However, ER is a proven factor that can trigger the onset and progression of KC through several mechanisms, including stimulation of inflammation.^[8]^ Children with KC are more likely to be boys and eye rubbers, and to have associated allergic diseases.^[9]^ Most studies^[10]^ report a relationship between atopic diseases and ASD; however, the findings remain controversial.

Chronic ER is one of the most important risk factors for developing KC and is the only modifiable factor. Therefore, some forms of KC may be preventable. Early behavioral intervention for ER habit reversal may be of particular benefit in children and adolescents with ASD. The aim of this report is to present an illustrative example of a boy with high-functioning autism (HFA) who developed progressive KC after undergoing CXL, which most likely was caused by his ER habit associated with allergy and by repetitive behavior due to ASD symptoms. We assumed that KC treatment with CXL combined with behavioral modification for ER may effectively prevent KC progression in children with ASD. To the best of our knowledge, a potential relationship between KC and ASD has not been previously reported.

Written informed consent to present this case report was obtained from the childs parent and with assent from the patient. All procedures were performed in accordance with the Institutional Review Board regulations and adhered to the tenets of the Declaration of Helsinki.

## 2 Case report

A 14-year-old boy was referred to our clinic on June 26, 2017 for an ophthalmic examination because of asymmetric visual acuity reduction. Progressive keratoconus was suspected. He was previously diagnosed with high-functioning ASD. The ASD diagnosis was verified, based on the clinical criteria of the *International Statistical Classification of Diseases and Related Health Problems, 10th Revision* (ICD-10),^[11]^ and verified using the Autism Diagnostic Interview–Revised (ADI-R),^[12]^ which a trained child psychiatrist administered. Psychological function was assessed by a clinical psychologist. The patient had no cognitive delay.

**Figure 1:**
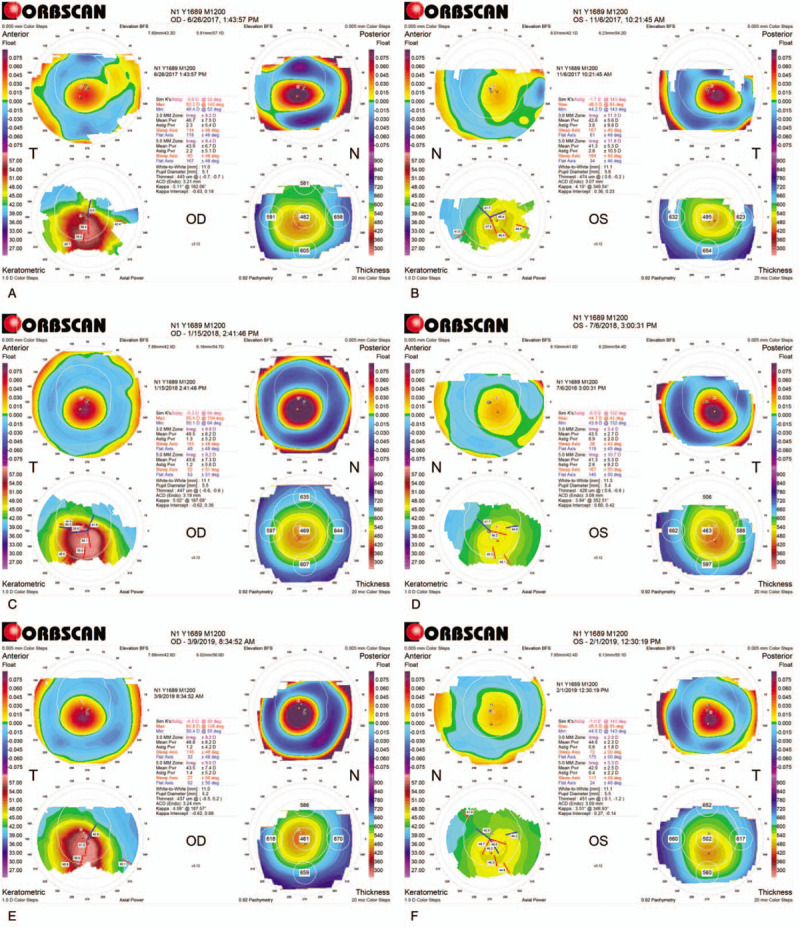
Corneal tomography at baseline and after the corneal cross-linking (CXL) procedure. Corneal tomography before the CXL shows a maximum keratometry (Kmax) value of 52.2D with a minimal corneal thickness (MCT) of 445 μm in the right eye (oculus dexter [OD]) (A). The Kmax value of the left eye (oculus sinister [OS]) is 46D with a MCT of 474 μm (B). Six months after CXL on the right eye, the Kmax increased to 55.4D with an increase in the posterior elevation float map (C). Six months after the CXL, the Kmax has decreased to 44.7D in OS (D). Twelve months after a repeat CXL procedure on the right eye, the Kmax has remained stable at 54.8D (E). Corneal tomography 13 months after the CXL on OS shows an unchanged Kmax of 45.5D, compared to baseline value (F).

The boy presented with deterioration of visual acuity because of an asymmetric increase in corneal astigmatism and myopia. At presentation, his uncorrected distant visual acuity was 20/50 and 20/20 in the right and left eye respectively (i.e., Snellen chart). Cycloplegic autorefraction values were – 0.25 DS with – 6.25DC × 65 in the right eye and-1.50DC × 100 in the left eye. Eyeglass-corrected visual acuity of his right eye was the same as the uncorrected acuity; pinhole visual equity was 20/40 and 20/20 in the right and left eye, respectively. A diagnosis of KC was confirmed by corneal tomography (Orbscan IIz Corneal Tomographer (Orbtek; Baush and Lomb, Salt Lake City, UT, USA) (Fig. 1A and B).

The child also presented with a history of intermittent exotropia, mostly at distant fixation. Measurements showed consistently poor control at near distance, with short periods of orthotropia at the 1-m distance, convergence insufficiency up to 15 cm, and exotropia of 40 PD distance fixation. The near deviation was easily dissociated and the patient did not spontaneously re-fuse. The left eye was strongly dominant. Random dot stereopsis (i.e., Lang I test) was negative, and Fly test could not be estimated because he was dissociated with polarizing glasses.

The slit-lamp examination showed both corneas were clear with no signs of Vogt striae. There was no evidence of papillary reaction on the tarsal conjunctiva. Intraocular pressure was within normal range in both eyes; however, the child involuntarily squeezed his lids, necessitating the examiners holding the eyelids open during Goldman applanation tonometry.

The child manifested avoidance of eye contact, limited verbal communication, reduced attention span, and moderate hypersensitivity to light. Communication with child was based on constant positive reinforcement and immediate verbal praise after each successful step during the examination. On interviewing the childs mother, a history of seasonal allergic rhinitis associated with itchy eyes became evident. However, the patients strong ER habit persisted throughout the year. The child rubbed his eyes with his knuckles in a circular motion. The boy, as well as his parents, was advised to abstain from his ER habit.

Without waiting for documentation of the progression of KC, CXL was performed on his right eye on July 27, 2017. Within 6 months, the same procedure was performed on his left eye on December 12, 2017. The child underwent standard epithelium-off CXL treatment under general anesthesia, as previously described.^[7]^ The central epithelium was first removed. An isotonic 0.1% riboflavin to 20% dextran solution (10 mg riboflavin-5-phosphate in 10 ml dextran solution) was then applied every 2 minutes over a 30-minute period. The central cornea was then exposed to ultraviolet (UV)-A irradiation using a UV light lamp (Intacs XL Corneal Cross-linking System; Addition Technology, Des Plaines, IL, USA) at 3 mW/cm^2^ for 30 minutes (i.e., 5.4 J/cm^2^ total energy dosage) with reapplication of isotonic riboflavin solution every 3 minutes to ensure saturation. At the end of surgery, a therapeutic soft contact lens was applied until complete re-epithelization of the cornea. After the operation, the patient was treated with ofloxacin eye drops 4 times daily (qid) for 1 week, fluorometholone eye drops qid with gradual taper over 1 month, and artificial tears qid for 6 months.

The child underwent a complete ophthalmic evaluation at all follow-up visits after CXL. Data analysis included uncorrected distant visual acuity and pinhole visual acuity, slit-lamp evaluation and corneal tomography values. We could not always obtain corneal tomography of both eyes at the same time because of reduced attention or because of increased sensitivity to light. The same examiner (SS) performed all follow ups. The corneal tomography results at baseline and after the CXL procedure (i.e., at 6 months and the 1-year follow-up) are presented in Figure 1.

**Figure 2:**
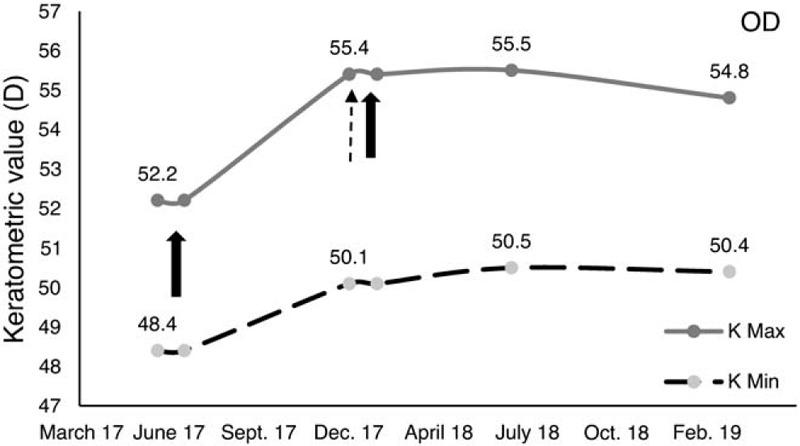
Changes in keratometric values in the right eye. At 6 months after corneal cross-linking (CXL) treatment, the Kmax value has increased by 3.2D. After retreatment with CXL in combination with behavioral intervention for eye rubbing (ER) reversal, the keratometric values for Kmax and Kmin remain stable at the 1 year follow-up. The solid arrow shows the timing of the CXL, whereas the dashed arrow shows the timing of the introduction of behavioral intervention for eye rubbing. Kmax = maximum keratometry, Kmin = minimum keratometry, OD = right eye.

At 6 months after the CXL treatment, a topographic examination of the right eye showed an increase in the maximum keratometry (Kmax) value by 3.2D from the baseline Kmax value (Fig. 1A and C and Fig. 2). The minimal corneal thickness was 447 μm (Fig. 1C). We advised that the patient undergo retreatment with CXL. It was performed as previously described. The childs parents noted that he did not fully comply with the advice to avoid his ER habit.

As an alternative approach, behavioral modification intervention for ER habit reversal, as previously described by McMoniee,^[13]^ was recommended. The four-stage behavioral modification approach to ER habit reversal includes being aware of the eye rubbing activity; finding alternative behavior responses (e.g., gentle lid hygiene or dry eye drops); developing high motivation (e.g., increased awareness of the association between abnormal ER and KC progression), and receiving social support by family members and friends.^[13]^

**Figure 3:**
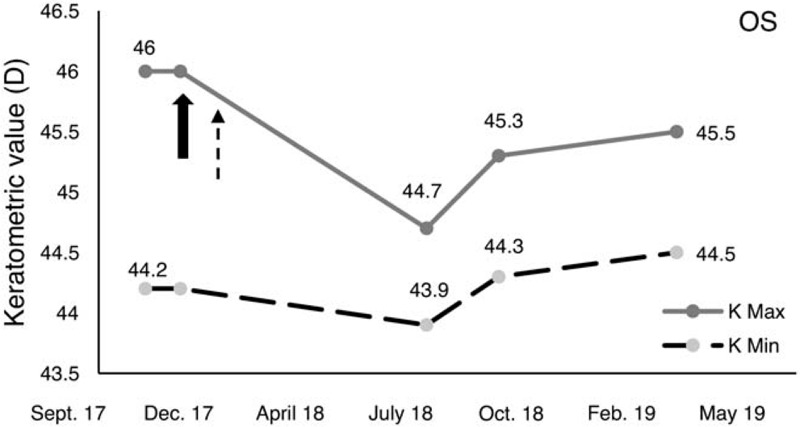
Changes in the keratometric values in the left eye. Treatment with CXL in combination with behavioral intervention for eye rubbing had stabilized the keratometric value at the 1 year follow-up. The solid arrow shows the timing of the CXL, whereas the dashed arrow shows the timing of the introduction of behavioral intervention for eye rubbing. Kmax = maximum keratometry, Kmin = minimum keratometry, OD = right eye.

At the last follow-up, 12 months after re-CXL procedure on his right eye, the keratometry readings remained stable (Fig. 1A and E and Fig. 2). No postoperative adverse effects occurred such as corneal haze nor epithelium healing delay. In the fellow eye, 13 months after standard epi-off CXL, keratometric values were not significantly changed in comparison with the preoperative readings (Fig. 1B and F and Fig. 3). Visual acuity remained stable in both eyes compared with baseline values. The boy also achieved and maintained full compliance with ER habit reversal after the retreatment with CXL. For a competing response, the boy used his hand to fan his eye; this action induced slight cooling on the surface of the eye. At home, refrigerated eye drops were also helpful as substitute for the ER habit. His parents followed the advice to be supportive in ER habit reversal.

## 3 Discussion

In this case report, we presented our experience with a 14-year-old boy with ASD who developed keratoconus in both eyes, most likely because of abnormal ER. This paper is aimed to increase awareness that ER as motor stereotype in children and adolescents with ASD may contribute to the development and progression of KC. We demonstrated that CXL in combination with behavioral interventions for ER habit reversal seems to prevent further keratoconus progression in a child with HFA. Optical management with contact lenses may be particularly challenging in children with ASD; therefore early management of KC in young patients with ASD is critically important.

Apart from allergy and atopy as the most common risk factors for ER in KC, emotional tension, psychosis, and compulsion may also be associated with ER habit.^[13]^ Bilateral self-induced KC associated with compulsive ER has been described in a patient with Tourettes syndrome.^[14]^ Corneal cross-linking in association with psychiatric treatment was effective for treating KC in patients with Tourettes syndrome.^[15]^

Restricted and repetitive patterns of behaviors (RBBs) are a core feature of ASDs.^[2]^ Motor stereotypy and self-injury such as eye rubbing are a “lower level” of RBBs.^[16]^ Eye pressing is commonly observed in children with ASD^[17]^; these individuals also may be at risk for developing KC. The literature indicates an increased prevalence of refractive errors and astigmatism in children with ASD,^[3,4,17–21]^ as presented in Table 1. However, further work would be valuable to estimate the etiology of the increased rate of corneal astigmatism in individuals with ASD. A higher prevalence of refractive errors and astigmatism in among pediatrics with genetic neurological and developmental disorders such as Down syndrome and cerebral palsy was primarily attributed to a failure in the normal process of emmetropization.^[22,23]^ At the same time, Down syndrome is a well-known risk factor for KC, most likely because of an ER habit, because a genetic correlation has not been confirmed.^[6]^ Clinical and tomographic screening of selected populations would provide a potential for timely diagnosis and subsequent treatment of KC. Godefrooij et al^[24]^ have recently demonstrated a 5- to 10-fold higher prevalence of KC, compared to previously reported figures (e.g., 265 cases per 100,000); this finding is the result of earlier and more advanced KC detection with corneal tomography.^[24]^

**Table 1:**
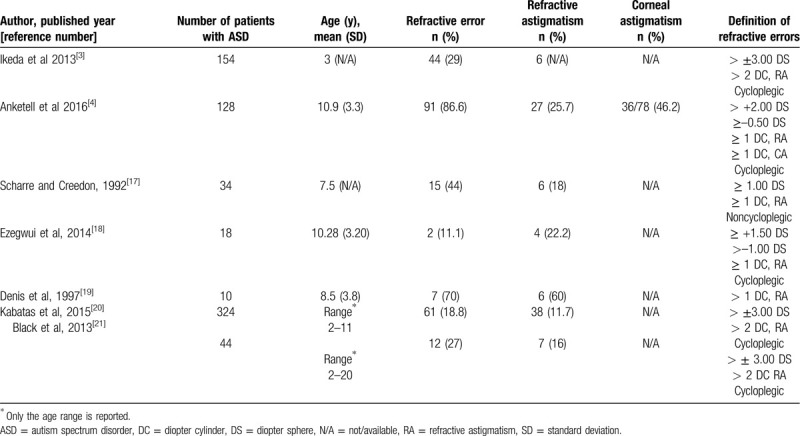
Refractive error in children with ASD in different studies.

Conducting an accurate tomography can be challenging in young patients with low-functioning ASD who may not fully cooperate. However, patients with HFA generally do comply with clear advice. Developing a rapport is critical for successfully performing different medical examinations in children with ASD. In our patient, the same examiner performed all procedures.

In a recent prospective case control study, manifest strabismus was estimated in 9% (11/124) of children with ASD, compared to 1.5% of age-matched typically developing controls.^[25]^ In our patient, the progression of keratoconus and reduced vision in his right eye interfered with the fusion and most likely contributed to deterioration of pre-existing intermittent exotropia. Prolonged visual deprivation of uncorrected poorer eye in some adult patients with asymmetric KC may cause a loss of stereopsis with manifest exotropia.^[26]^

Standard CXL is safe and the most effective way of KC management in children.^[27]^ However, because of the reduced corneal stiffness, CXL in children is not as effective as it is in adults. Progression of KC after CXL treatment may also be related to a persistent ER habit.^[27]^ Our patient likewise showed signs of KC progression at 6 months after the standard CXL procedure. He underwent retreatment. Advising a patient to abstain from ER is usually inefficient for a substantial number of patients.^[13]^ This situation also occurred for our patient. A behavioral modification approach to controlling an ER habit, as described by McMonnnies,^[13]^ may be a useful alternative for these patients, including individuals with ASDs. Symptoms of ASDs are behavior-related; therefore, behavior modification is a primary intervention in this disorder.

Early behavioral interventions are likely to provide a positive effect and to improve RBBs in children with ASD.^[28]^ Therefore, behavioral modification intervention could be adapted prophylactically for children with ASD who may be at risk for development of or progression of KC because of frequent and vigorous ER related to RBBs or allergy. In the presented case, we also found that this method had the potential to motivate the 14-year-old child with HFA to achieve and maintain full compliance with ER habit reversal, after retreatment with CXL.

In conclusion, children with ASD may be at higher risk for developing keratoconus because of an ER habit associated with atopic diathesis and because of repetitive behavior due to ASD symptoms. Careful attention should be administered to proactive screening for keratoconus in this selected population group. The condition has lifelong effects for the individual; thus, early behavioral intervention for ER and management of KC with CXL help preserve visual acuity and improve outcomes.

## Acknowledgments

We would like to thank Editage (www.editage.com) for English language editing.

## Author contributions

**Conceptualization:** Svetlana Stanojlovic, Milica Pejovic Milovancevic.

**Data curation:** Svetlana Stanojlovic, Milica Pejovic Milovancevic, Branislav Stankovic.

**Formal analysis:** Svetlana Stanojlovic.

**Investigation:** Svetlana Stanojlovic, Milica Pejovic Milovancevic.

**Methodology:** Svetlana Stanojlovic, Milica Pejovic Milovancevic, Branislav Stankovic.

**Supervision:** Svetlana Stanojlovic, Milica Pejovic Milovancevic, Branislav Stankovic.

**Writing – original draft:** Svetlana Stanojlovic, Milica Pejovic Milovancevic, Branislav Stankovic.

**Writing – review & editing:** Svetlana Stanojlovic, Milica Pejovic Milovancevic, Branislav Stankovic.
